# Tombolas and Their Critics

**Published:** 1919-10-25

**Authors:** 


### TOMBOLAS AND THEIR CRITICS.

It is proper to follow our discussion in these pages of hospital policy in regard to tombolas by the news that, as a correspondent puts it, the governors of the Norfolk and Norwich Hospital have been roundly criticised for spreading the "lust of gambling" by their recent tombola, which yielded the handsome sum of £13,000 towards the big appeal (for £30,000) for the orthopædic department which Sir Hamilton Ballance is organising at the hospital. Among the critics was the Bishop of Norwich. At the latest meeting of the governors Dr. S. Barton replied humorously and with spirit to these critics. Having pointed out that in legacies and donations there had been received a little over £2,000, he mentioned that to Dr. and Mrs. Burton Fanning, who lost their eldest son and daughter during the war, the governors wished to express their deep sympathy. Dr. and Mrs. Burton Fanning wished to perpetuate the memory of their daughter Beris, who had worked eagerly in the soldiers' wards in the early part of the war. They had given the handsome donation of £750 to found a Nurses' Aid Fund, the income of which was to be used for any of the hospital nursing staff requiring financial or other help. Referring to the scheme for the new Orthopædic Department, Dr. Barton said that as the result of their big appeal for £30,000 they had been promised £7,000 as donations, and had handed over to the hospital £13,000 obtained by the tombola. Many friends of the hospital freely gave their assistance for weeks before the great day of the tombola. For several nights the work lasted until after ten o'clock. There was not one penny of the ticket money that did not come in full to the Appeal Fund; all the expenses had been defrayed by a fête. "I am sorry to say," said Dr. Barton, "that our big effort has not given total satisfaction. We have been called law-breakers, the degradation of our methods has been deplored, our expedients have been unworthy, and we have influenced thousands with the lust of gambling, and so forth. On thinking over this list of crimes we may find comfort for ourselves by reading the legend of the 'Jackdaw of Rheims.' You remember the Cardinal's curse:

"'He cursed him in sitting, in standing, in lying;
He cursed him in walking, in riding, in flying;
He cursed him in living, he cursed him in dying;
Never was heard such a terrible curse.
But what gave rise to no little surprise,
Nobody seemed one penny the worse.'"

A quotation which was greeted with much laughter. "But the C.O.s outside their obsession have been most liberal, having sent me four substantial cheques, so we must forgive them as we hope to be forgiven."

Canon Pelham remarked that a great debt of gratitude was owed to those who organised the tombola. Those friends of the hospital only had the good of the hospital in view, and they had satisfied themselves that the tombola was a perfectly harmless piece of machinery. Yet at the same time he was convinced that the Board of Management made a mistake in sanctioning the tombola. He knew very well how extremely difficult it was to draw a dividing line between what was expedient and what was inexpedient. In every department of hospital work the highest possible ideals were encouraged. Work for the hospital was a pure delight. On the other hand, the appeal of a tombola was not to the highest and best instincts of the nation, but rather to the lower instincts. Therefore he hoped that in the future they would never sanction anything which seemed to sin against the characteristic spirit of that great institution.

